# Preliminary analysis of the metabolic and physical activity profiles of mice lacking the *slc43a3*-encoded equilibrative nucleobase transporter 1

**DOI:** 10.1371/journal.pone.0323853

**Published:** 2025-08-21

**Authors:** Aaron L. Sayler, James R. Hammond

**Affiliations:** Department of Pharmacology, Faculty of Medicine & Dentistry, University of Alberta, Edmonton, Alberta, Canada; University of Texas Southwestern Medical Center, UNITED STATES OF AMERICA

## Abstract

*SLC43A3* encodes for a membrane transporter selective for purine nucleobases (equilibrative nucleoside transporter 1; ENBT1). Adenine, an endogenous substrate for ENBT1, plays an important role in many biochemical and physiological processes, including cellular energy metabolism. To investigate how the loss of ENBT1 impacts these processes, we generated a *slc43a3*-null (global; KO) mouse model. Metabolic function, physical activity, and food and water consumption were assessed in male and female wild-type (WT) and KO mice (age 10–12 weeks) for a 60-hour period (12 hr light/dark cycle). Blood pressure and heart rate of each group of mice were also assessed using a rodent tail cuff method. Male KO mice showed a significant increase in metabolic activity relative to male WT mice. Male KO mice also displayed a significant decrease in rearing activity and blood pressure. Female KO mice did not show the same changes in metabolic and physical activity as the males, but did display a significant 4-hour negative change in diurnal rhythm phase in the metabolic and activity measures that was not seen for the male KO mice. It may be concluded that loss of *slc43a3*-encoded ENBT1 impacts numerous measures of activity in mice, with female mice impacted differently than male mice. This may reflect disruption of purinergic processes associated with energy metabolism coincident with changes in cellular adenine availability.

## Introduction

*SLC43A3* encodes for a membrane transporter selective for purine nucleobases (equilibrative nucleoside transporter 1; ENBT1) [1,2]. Adenine, an endogenous substrate for ENBT1, plays an important role in many biochemical and physiological processes, including serving as a precursor for the intracellular production of adenine nucleotides [3,4]. Adenine nucleotides are integral to cellular energy metabolism and adenosine has a host of biological regulatory functions including being involved in vaso-regulation and neuronal modulation [5]. Therefore, it is not unreasonable to expect that the genetic deletion of *slc43a3* in mice would have a significant impact on cellular purine availability and biological functions that are regulated by purine nucleosides and nucleotides. We have recently published on the creation and use of a *slc43a3*-null (global) mouse model to assess the role of the encoded transporter in the absorption of orally administered 6-mercaptopurine [6]. These *slc43a3*-null mice were found to be viable with no obvious defects in gross morphology. We now report on an analysis of the metabolic energy profile, food and water consumption, mobility, and cardiovascular impact of the loss of *slc43a3* in these mice.

## Materials and methods

*Slc43a3*-null (KO) and wild-type (WT) C57BL/6J mice were both obtained in-house via *slc43a3*^+/-^(heterozygous) pairings as described previously [6]. Food and water were provided ad libitum and standard housing was used. Mice were euthanized at the end of the study by carbon dioxide overdose. All procedures were performed by personnel with specific training in rodent handling to reduce stress and alleviate suffering in the animals. All animal work was conducted according to the Canadian Council on Animal Care standards using protocols (AUP00002022) approved in writing by the Animal Care and Use Committee (Health Sciences) of the Faculty of Medicine & Dentistry, University of Alberta.

A comprehensive lab animal monitoring system (CLAMS-HC, Columbus Instruments, Columbus, OH) was used in tandem with Columbus Instruments Oxymax Lab Animal Monitoring System software (Cardiovascular Research Centre, University of Alberta) to measure total horizontal volitional movement (X-activity), ambulatory activity (multiple consecutive beam breaks), Z-activity (rearing), food intake, water intake, volume of O_2_ consumption (VO_2_), volume of CO_2_ production (VCO_2_), respiratory exchange ratio (RER) and energy expenditure. Mice had access to standard chow (5L0D - PicoLab® Laboratory Rodent Diet) and demineralized water and were singly housed at ~23° C (range 22.4°-23.8°) for these analyses. Eight mice of each sex and genotype (WT, KO) were assessed at 10–12 weeks of age. Mice were acclimated to the metabolic cages for 24 hours, and the above noted parameters were then measured continuously (every 1–3 minutes) over 60 hours. LED lighting configuration was 12-hour dark (hue, saturation, brightness: 0, 100%, 50%), 12-hour light (hue, saturation, brightness: 0, 0%, 100%), cycling over a 3-day period starting at 7:00 pm (beginning of dark cycle) on the first day to 7:00 am on the third day. For analyses, the data obtained were binned by hour (averaged for VO_2_, CO_2_, RER, and energy expenditure; summed for activity measurements and food and water consumption). Cosinor analysis was conducted to determine the mesor (midline estimating statistic of rhythm), amplitude of diurnal fluctuations, and phase for each parameter using the FFT NLLS method [7] for the highly rhythmic metabolic data, and the MFourFit method for the food/water consumption and activity data (which showed less robust rhythmicity), both with linear detrending and no constraints (biodare2.ed.ac.uk) [8,9]. This cosinor/phase analysis was conducted on individual animals to assess within group variation and correlations between measured parameters. Comparisons between different genotypes and sexes were done using group means and standard errors with statistical differences assessed using one-way ANOVA with Tukey’s post test, P < 0.05 (GraphPad Prism v10.4).

Cardiovascular parameters (systolic and diastolic blood pressure, heart rate) were measured using a CODA^®^ High Throughput (Kent Scientific, Torrington, USA) non-invasive blood pressure measurement system designed specifically for rodents, by the Cardiovascular Research Centre, University of Alberta. A total of 8 WT mice and 8 KO mice of each sex were analyzed at 10–12 weeks of age. Animals were placed in the CODA^®^ Animal Holders wearing the CODA^®^ High Throughput occlusion cuffs and volume-pressure recording cuffs on a heating pad (35°C) and covered with an opaque insulating sheet for calming effect to assist with attaining normal physiological blood flow. Cardiovascular parameters were recorded between 7:00 and 8:00 am once a minute for a total of 15 minutes. All animals were acclimated in this facility for 15-minute periods on 3 successive days preceding the experiments to familiarize them with the procedure and reduce stress-induced cardiovascular anomalies.

## Results

The data obtained from these studies (S1 Table) were assessed for both the magnitude of the change resulting from loss of *slc43a3*, and the effect of this gene deletion on diurnal rhythm. Differences in the parameters between male and female mice were also evaluated. The deletion of *slc43a3* had no significant impact on the overall body mass for both male (WT: 26.0 ± 0.3 g, KO: 26.5 ± 0.5 g) and female (WT: 21.6 ± 0.2 g, KO: 21.8 ± 0.4 g) mice. Nor were there any other gross abnormalities observed in the KO mice over the 12-week life span used for this study.

**Within group individual animal analyses:** Data obtained from cosinor analysis for each animal used in this study are shown in S2 Table. The cycle length (period) in each case was not different from 24 hours, reflecting entrainment to the 12-hour dark/light environment. When assessing within group variability, the only observation that stands out is that female KO mice showed more variability in the metabolic and activity measurements compared with the other genotype/sex groups. Correlation analysis was conducted within each genotype/sex group to determine the relationship between the measured parameters. Pearson coefficients from these correlations are shown in S2 Table along with statistical significance indicators (P < 0.05). For analysis of these correlations, the measured parameters were grouped into metabolic rate indicators (VO_2_, CO_2_, energy expenditure), physical activity (X-activity, ambulatory activity, Z-activity), and food/water consumption. RER was not included in the metabolic function grouping due to the lack of body composition (lean/fat) data, making interpretation of changes in this parameter problematic. Male WT mice showed no significant correlations between metabolic parameters, activity measurements, and food/water consumption in any of the phase analysis parameters. In contrast, male KO mice showed a significant positive correlation in phase between X-activity and energy expenditure. There was also a significant positive correlation in cycle amplitude between VCO_2_ and X-activity, and a positive correlation in cycle amplitude between energy expenditure and water intake. Furthermore, there was a significant correlation in mesor between VO_2_ and VCO_2_ and Z-activity. Female WT mice, unlike the male WT mice, showed a strong positive correlation between many of the measured parameters. Specifically, female KO mice showed a significant correlation in phase between metabolic activity and rearing (Z-activity). There was also a significant correlation in phase between the physical activity measurements and food and water intake. Female WT mice also showed a significant correlation in cycle amplitude between metabolic activity and rearing and food/water intake. Female WT mice were similar to the male KO mice in that there was a significant correlation in mesor between Z-activity (rearing) and metabolic rate. Female KO mice had a strong positive correlation in phase between all measures of metabolic function and all physical activity measurements, suggesting that while there was significant variability between individual mice is this group, each animal retained its own unique phase with respect to metabolic activity and physical activity. The only apparent correlation with respect to cycle amplitude was between the metabolic function measurements and water intake, and there were no correlations between any of the measurement groups in the mesor of the female KO mice.

**Fig 1:**
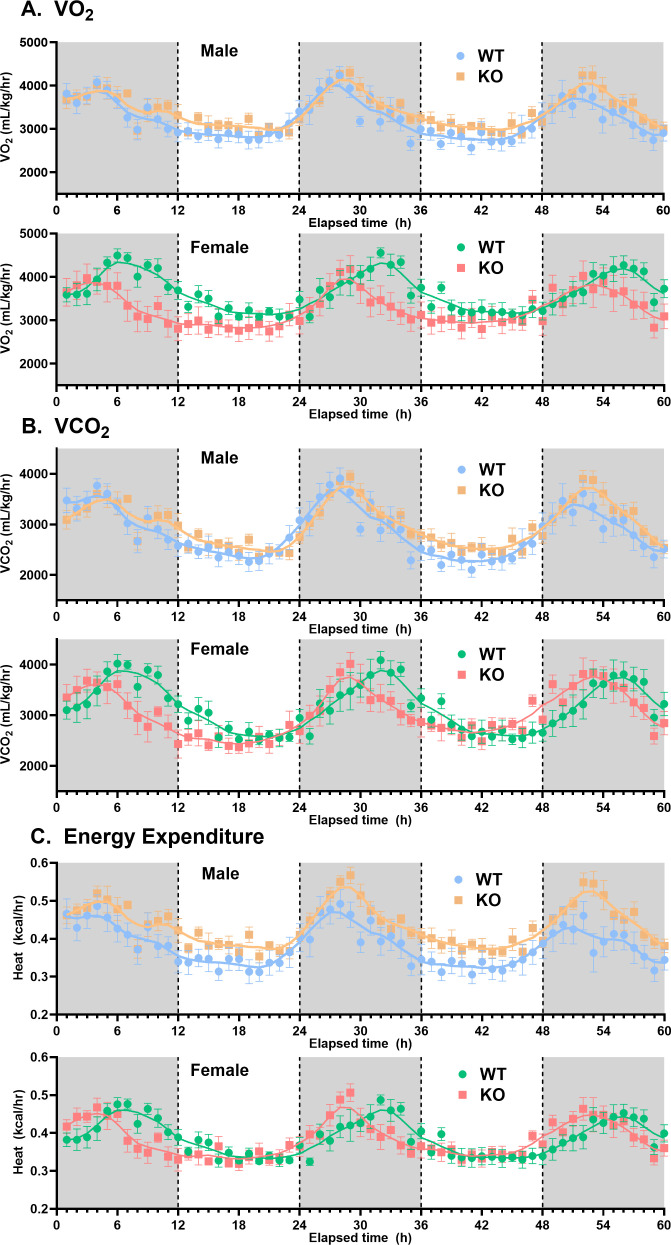
Effect of *slc43a3* deletion on metabolic performance parameters in male and female WT and KO mice. Using an automated Columbus Lab Animal Monitoring System (CLAMS), oxygen consumption (VO_2_) and carbon dioxide generation (VCO_2_) were measured over 60 h (12 h dark/light), starting at 19:00 h (initiation of dark phase). The values for respiratory exchange ratio (RER) and energy expenditure were derived from these data. Each point is the mean ± SEM from 8 mice. The lines represent LOWESS curve fits to the data using a 20-point smoothing window (GraphPad Prism v10.2).

**Fig 2:**
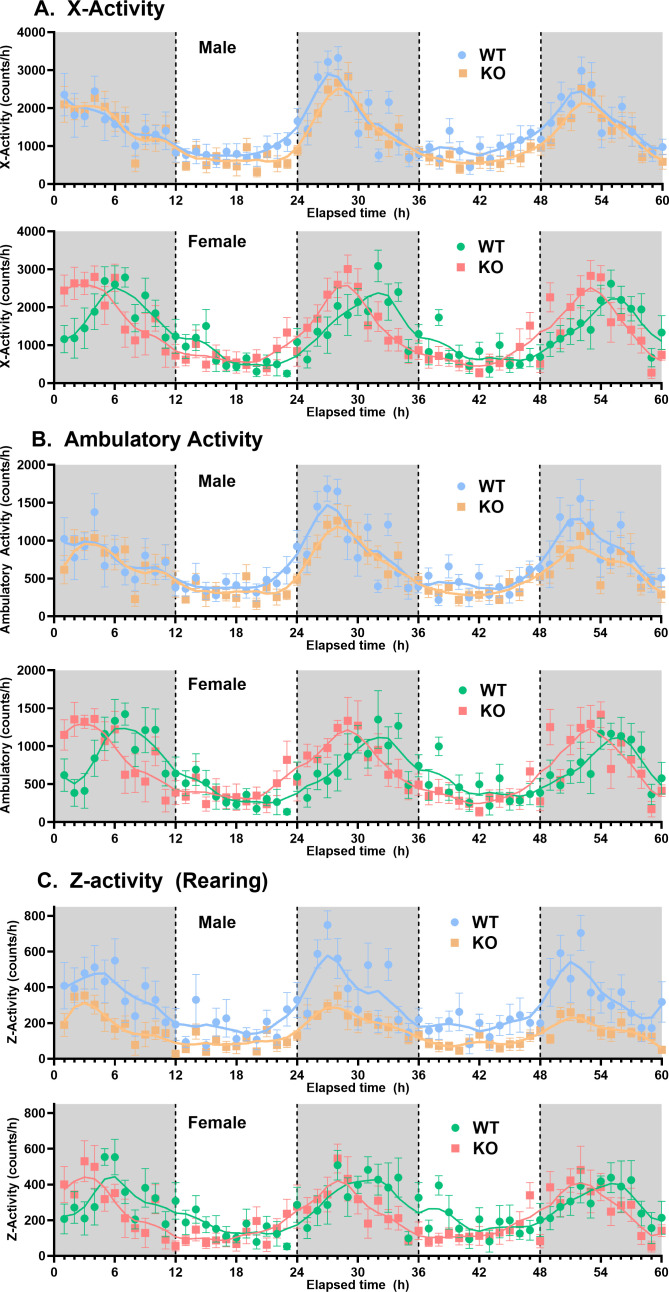
Effect of *slc43a3* deletion on physical activity of male and female WT and KO mice. Using an automated Columbus Lab Animal Monitoring System (CLAMS), the total number of horizontal beam breaks (X-Activity), the number of consecutive horizontal beam breaks (Ambulatory Activity), and the number of elevated beam breaks (Z-Activity, representing rearing activity of the mouse) were measured continuously over 60 h (12 h dark/light), starting at 19:00 h (initiation of dark phase), and binned to obtain the beam breaks (counts) per **h.** Each point is the mean ± SEM from 8 mice. The lines represent LOWESS curve fits to the data using a 20-point smoothing window (GraphPad Prism v10.2). The area under the curve for these data sets (Total movement counts) are shown in Table 1.

**Fig 3:**
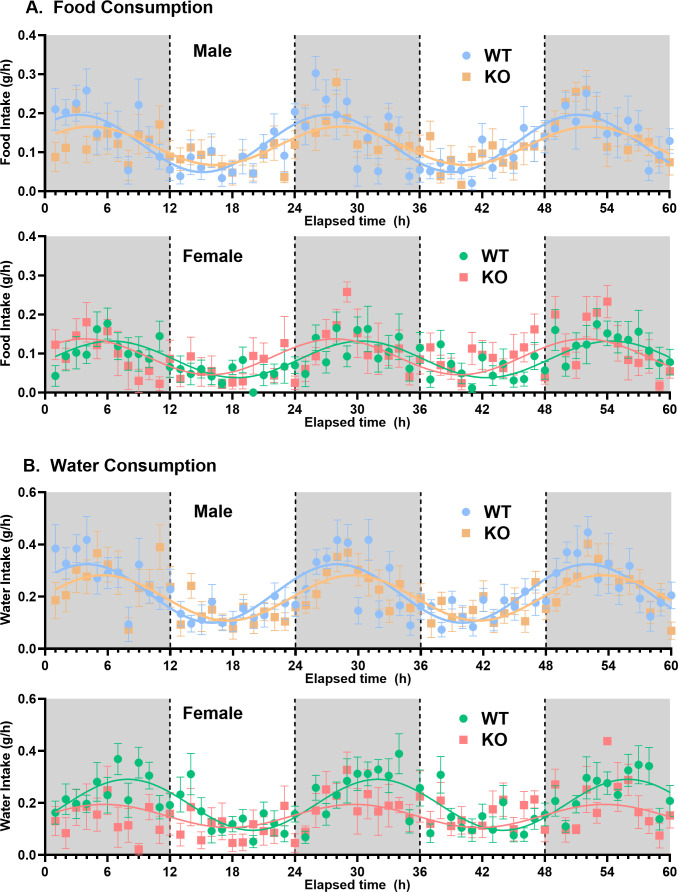
Effect of *slc43a3* deletion on food and water consumption in male and female WT and KO mice. Using an automated Columbus Lab Animal Monitoring System (CLAMS), the amount of food and water consumed were measured every 2-3 minutes over a period of 60 h (12 h dark/light), starting at 19:00 h (initiation of dark phase), and binned to obtain g/h. Each point is the mean ± SEM from 8 mice. The lines represent LOWESS curve fits to the data using a 20-point smoothing window (GraphPad Prism v10.2). The area under the curve for these data sets (total food and water consumed) are shown in Table 1.

**Genotype/sex group comparisons:** For comparisons among sex/genotype groups, averaged data (±SEM) were used (Table 1). In terms of changes in metabolic activity, female WT mice had a significantly higher (by approximately 25%) overall average (mesor) VO_2_ and VCO_2_ compared with male WT mice, but deletion of *slc43a3* abolished this sex difference (Fig 1, Table 1). The female KO mice also showed an approximate 20% decline, relative to female WT mice, in the magnitude of the fluctuations (cosine amplitude) in VO_2_ over the 24-hour cycle (Fig 1, Table 1). Due to the relatively parallel changes in VO_2_ and VCO_2_ in these subject groups, there were no differences in RER between the WT and KO mice (Table 1). Loss of *slc43a3* also led to a significant 14% increase in metabolic energy expenditure in the male mice, but not in the female mice (Table 1). This increase in energy expenditure in the male KO mice was accompanied by a significant decrease in all physical activity measures (X-activity, ambulatory, and rearing) in the male KO mice that was not seen for the female KO mice (Fig 2, Table 1). The decrease in rearing activity was particularly dramatic in the male KO mice with close to a 40% decline relative to the WT mice. With respect to feeding and drinking behaviour, the only change noted between WT and KO mice was a decrease in the average water intake in the female KO mice, relative to female WT mice (Fig 3, Table 1).

**Table 1:** Summary of measured parameters.

Measure	Male WT	Male KO	Female WT	Female KO
**VO**_**2**_ **(ml/kg/h)**				
Mesor^a^	**2984 ± 157** ^**# d**^	3387 ± 111*	3581 ± 77	3438 ± 99
Amplitude^b^	506 ± 52	481 ± 26	654 ± 27	533 ± 50*
Phase^c^ (h)	**3.75 ± 0.33** ^**#**^	5.01 ± 0.47	7.56 ± 0.72	4.71 ± 0.88*
**VCO**_**2**_ **(ml/kg/h)**				
Mesor	**2367 ± 168** ^**#**^	2956 ± 94*	3092 ± 99	2981 ± 102
Amplitude	579 ± 56	555 ± 24	747 ± 28	589 ± 69
Phase (h)	**4.28 ± 0.42** ^**#**^	5.87 ± 0.40	7.81 ± 0.77	5.39 ± 0.85*
**RER**				
Mesor	0.871 ± 0.011	0.869 ± 0.010	0.856 ± 0.012	0.866 ± 0.013
Amplitude	0.05 ± 0.01	0.05 ± 0.01	0.06 ± 0.01	0.06 ± 0.02
Phase	6.24 ± 1.61	7.18 ± 0.93	7.99 ± 1.13	8.02 ± 1.39
**Energy Expenditure (kcal/h)**				
Mesor	0.377 ± 0.03	0.431 ± 0.012*^†^	0.380 ± 0.008	0.370 ± 0.010
Amplitude	0.07 ± 0.01	0.07 ± 0.01	0.07 ± 0.01	0.06 ± 0.01
Phase (h)	**3.91 ± 0.37** ^**#**^	5.33 ± 0.38	7.64 ± 0.75	4.84 ± 0.96*
**Food (g/h)**				
Mesor	**0.122 ± 0.011** ^**#**^	0.127 ± 0.004	0.083 ± 0.012	0.120 ± 0.008
Amplitude	0.09 ± 0.01	0.07 ± 0.01	0.07 ± 0.01	0.07 ± 0.01
Phase (h)	4.99 ± 1.27	7.45 ± 1.69	9.85 ± 1.61	12.11 ± 2.78
Total Food Consumed (g)	7.6 ± 0.6	7.3 ± 0.6	5.4 ± 0.6	5.7 ± 0.6
**Water (ml/h)**				
Mesor	0.214 ± 0.016	**0.204 ± 0.007^†^**	0.193 ± 0.008	0.167 ± 0.009*
Amplitude	0.12 ± 0.02	0.12 ± 0.01	0.13 ± 0.01	0.08 ± 0.02*
Phase (h)	**4.47 ± 0.99** ^**#**^	5.42 ± 1.13	8.64 ± 1.08	9.33 ± 3.33
Total Water Consumed (ml)	13.1 ± 0.9	12.3 ± 0.9	12.1 ± 0.9	9.3 ± 0.8
**X-Activity (counts/h)**				
Mesor	1317 ± 141	1102 ± 56*	1344 ± 154	1199 ± 78
Amplitude	**907 ± 62** ^**#**^	844 ± 79	1238 ± 115	982 ± 118
Phase (h)	**3.47 ± 0.72** ^**#**^	4.12 ± 0.68	7.83 ± 0.76	3.93 ± 0.67*
Total Counts (/1000)	82.9 ± 5.0	71.5 ± 4.4	78.6 ± 5.5	78.7 ± 5.0
**Ambulatory (Walking) (counts/h)**				
Mesor	710 ± 83	589 ± 39*^†^	731 ± 120	682 ± 49
Amplitude	551 ± 55	427 ± 59	634 ± 100	567 ± 67
Phase (h)	**3.91 ± 0.46** ^**#**^	4.62 ± 0.62	8.47 ± 0.71	3.86 ± 0.68*
Total Counts (/1000)	41.8 ± 2.7	35.1 ± 2.3	39.3 ± 2.9	40.2 ± 2.6
**Z-Activity (Rearing) (counts/h)**				
Mesor	**349 ± 39** ^**#**^	214 ± 11*^†^	423 ± 79	337 ± 40
Amplitude	201 ± 25	109 ± 20*	261 ± 37	270 ± 36
Phase (h)	3.30 ± 0.83	5.60 ± 1.95	7.02 ± 1.30	3.61 ± 0.61*
Total Counts (/1000)	17.7 ± 1.3	9.0 ± 0.6*	15.5 ± 1.2	13.1 ± 1.0

^a^Mesor of a cosine wave function, derived from analyses of individual mice (GraphPad Prism v 10.2).

^b^Amplitude of fluctuation, derived from cosinor analyses of individual mice (BioDare2).

^c^Diurnal phase from the start of the dark cycle, derived from cosinor analyses of individual mice (BioDare2).

^d^Mean ± SEM, N = 8.

*Significant difference between WT and KO of same sex (Two-way ANOVA with Tukey’s post-test, P < 0.05).

# Significant difference between male and female WT (Two-way ANOVA with Tukey’s post-test, P < 0.05).

^†^Significant difference between male and female KO (Two-way ANOVA with Tukey’s post-test, P < 0.05).

**Fig 4:**
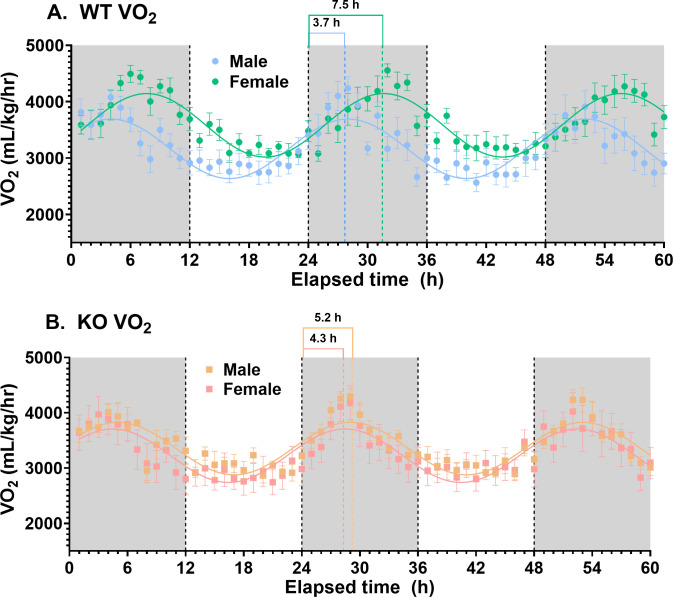
Effect of *slc43a3* deletion on the diurnal cycle of oxygen consumption (VO_2_) in male and female mice. Panel A and B show data from WT and KO mice, respectively, with a cosine wave function (parameters from BioDare2 analyses) fitted to each data set. Note that these data are from Fig 1A, re-plotted to illustrate the shift in diurnal rhythm in the female mice upon deletion of *slc43a3*. Each point represents the mean ± SEM from 8 mice.

Another highly significant effect of deletion of *slc43a3* was a change in phase for the diurnal cycles of the female mice. This was apparent in the metabolic VO_2_, VCO_2_, and energy expenditure profiles, as well as in all of the activity measurements (Table 1, Figs 1–2, and 4); female WT mice had nocturnal phase peaks of 7–10 hours, depending on measured parameter, while the female KO mice were more consistent with a peak occurring 4–5 hours after the start of the dark phase. This is also illustrated visually in Fig 4 for the VO_2_ data set, where it Is apparent that the loss of *slc43a3* eliminates the sex difference in the diurnal cycle. There was a similar trend in the food and water consumption in the female mice, but due to the greater variability in these data, the sample size used in the current study did not have the statistical power to detect small differences (Fig 3, Table 1). This highlights a limitation of this study in that the sample size of 8 mice is less than that typically needed for robust statistical analysis of these types of studies. A similar shift in phase was not seen for the male KO versus WT mice. If anything, metabolic activity in the male KO mice tended to shift to a later nocturnal phase peak (~6 hours) relative to male WT mice (~4 hours) (Table 1). This shift in the diurnal rhythm of the female KO mice eliminated the difference in diurnal rhythm that was evident between the male and female WT mice (Fig 4).

**Fig 5:**
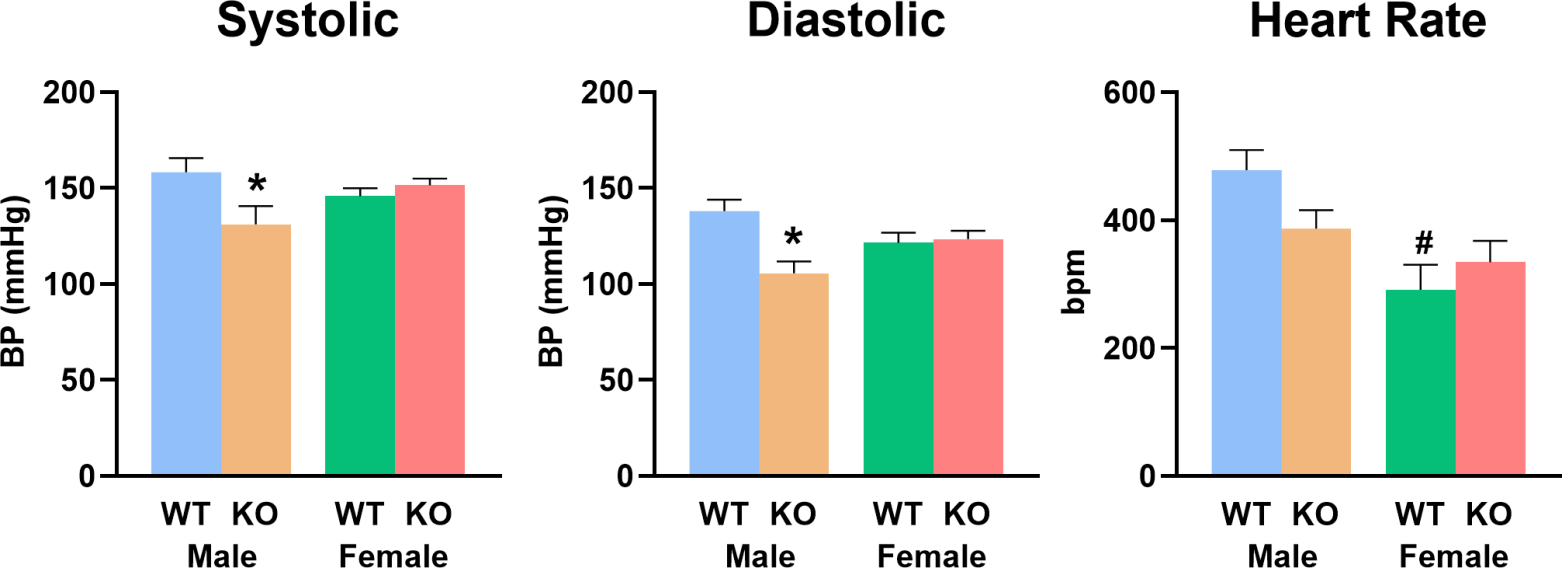
Effect of *slc43a3* deletion on resting blood pressure (systolic and diastolic) and heart rate of male and female WT and KO mice. Cardiovascular function was measured using a rodent tail cuff apparatus during the light-phase of the diurnal cycle. * Indicates a significant difference between WT and KO mice of the same sex; ^#^ Indicates a significant difference between male and female mice of the same genotype (Two-way ANOVA with Tukey’s post-test, P < 0.05).

Given the well-established roles of purines in the regulation of cardiovascular function [10], we also compared the WT and KO mice with respect to their blood pressure and heart rate. Male KO mice showed a significant decrease (by about 20%) in both systolic and diastolic blood pressure, and a trend towards a decrease in heart rate relative to the male WT mice (Fig 5). In contrast, female WT and KO mice were not different with respect to their cardiovascular parameters.

## Discussion

The metabolic and behavioural characteristics of the mice used in this study were consistent with previously published data on young adult C57BL/6J mice [11]. While no gross abnormalities were observed in the KO mice, analyses of metabolic function and activity profiles showed significant impacts of the loss of *slc43a3*. Both VO_2_ and VCO_2_ increased in the male KO mice relative to male WT mice with no corresponding change in RER. The metabolic activity of female mice was not similarly impacted by the loss of *slc43a3,* highlighting a sex-specific effect in this regard. While speculative in the absence of mouse body composition data (lean/fat ratios), this does suggest that there is an increase in metabolic rate in the male KO mice with no change in the relative contribution of carbohydrates and fat utilization. In this regard, it is noteworthy that there were no differences in overall body weights between the WT and KO mice of either sex, and previous work has shown that there are no observable differences between WT and KO mice in major organ weights or fat content upon dissection [6]. *Slc43a3* encodes for an equilibrative nucleobase transporter that is involved in the salvage of purine nucleobases (e.g., adenine) by cells [1,2]. Given the established roles of purine nucleotides in cellular energy metabolism [12,13], and the fact that adenine salvage can supplement AMP production via adenine phosphoribosyltransferase (APRT) [14], it is not surprising that the loss of the primary adenine salvage transporter (ENBT1) would have an attenuating impact on oxygen consumption (VO_2_) and carbon dioxide generation (VCO_2_) which reflect overall cellular metabolic activity in the mice. The sex specific nature of this effect is intriguing though. We have previously shown that male and female WT mice have similar levels of expression of *slc43a3*, and that the loss of this gene does not impact the expression of other enzymes, including APRT, involved in purine metabolism, in either sex [6]. This leads to the interesting possibility of a relationship between sex hormones and ENBT1 transport activity in the regulation of metabolic activity as a target for future investigation.

The other very significant sex-specific difference observed was the diurnal phase difference between male and female WT mice for all of the parameters measured. Even more striking was the fact that deletion of *slc43a3* eliminated this difference between male and female mice (with the specific impact being on the female mice). While the molecular/cellular signalling pathways involved in the control of circadian rhythms are complex [15], there are several mechanisms by which disruption of the purine metabolic pathways (via reduced salvage of purine nucleobases by ENBT1) may impact these rhythms. This is particularly important considering that adenine is a metabolic precursor to adenine nucleotides. Indeed adenosine-receptor mediated signaling itself has been implicated in the regulation of the circadian clock [16]. Adenosine 3’,5’-monophosphate kinase (AMPK) phosphorylates the transcription factors PER and CRY that are integral to circadian clock control, thus promoting their polyubiquitination and subsequent degradation [17,18]. AMPK activity is regulated by the ratio of AMP:ATP in cells [19], which is sensitive to purine nucleoside/nucleobase levels [20]. Furthermore, calcium/cAMP response elements have been found in the promoters of several clock genes, and cAMP-dependent signaling has been proposed as a core component of the mammalian circadian pacemaker [21]. Adenosine can regulate intracellular calcium levels [22,23] and is a precursor to cAMP. Our finding that it is specifically the female diurnal rhythm that is changed in the *slc43a3-null* mice, implies that sex hormones, such as estrogen, may play a role in this. This does, however, highlight a limitation of the current study in that female mice were not monitored/controlled for stage of estrus cycle. Therefore, hormonal levels might be expected to vary significantly between mice. However, it is also notable that there was no difference in variability in the data obtained in the female mice relative to the male mice suggesting that this was not a complicating factor in this study. While sex differences in circadian rhythms have been well documented [24,25], there is notably less information in the literature regarding the underlying mechanisms for these differences. While still speculative, a possible linkage between purine metabolism, estrogen, and diurnal rhythm may involve AMPK. The observed disruption of metabolic activity in the female mice upon loss of *slc43a3* may impact AMPK activity in the female mice specifically leading to changes in diurnal rhythm control. It is also noteworthy that AMPK has been shown to be regulated by estrogen [26], which may also be a factor in the sex-specific differences noted in the present study.

Another sex-specific difference observed was the significantly decreased level of physical activity seen in the male *slc43a3*-KO mice, relative to the male WT mice, particularly with respect to their rearing activity. This is interesting in light of the significant positive correlation in the VO_2_/VCO_2_ values and rearing activity in the male KO group that was not seen in the male WT mice. This suggests that the male KO mice may require more metabolic work to support their rearing activity, possibly reflecting respiratory or cardiovascular dysfunction. This theory is supported by the finding that there was a significant decrease in both systolic and diastolic blood pressure in the male KO mice, and an increase in overall energy expenditure relative to male WT mice. While interpretation is confounded somewhat by the fact that blood pressure was measured during the light phase and rearing activity occurred primarily during the dark phase, it has been reported that chronic low blood pressure in mice reduces their performance in rotarod tests [27], suggesting that problems with maintaining balance might also underlie the decrease the frequency/duration of rearing observed. Further interpretation requires additional analyses using free-roaming telemetry monitoring of blood pressure and positional information.

While mechanistic underpinnings of the changes observed require further investigation, the loss of *slc43a3* in mice clearly results in significant changes in metabolic activity in female mice and changes in cardiovascular function and physical activity measures in male mice. These data highlight the importance of the purine nucleobase transporter ENBT1 in the endogenous regulation of metabolic function.

## Supporting information

S1 Table Raw data. (XLSX)

S2 Table Phase analysis data and within group correlations. (XLSX)
